# The influence of venous admixture on alveolar dead space and carbon dioxide exchange in acute respiratory distress syndrome: computer modelling

**DOI:** 10.1186/cc6872

**Published:** 2008-04-18

**Authors:** Lisbet Niklason, Johannes Eckerström, Björn Jonson

**Affiliations:** 1 Department of Clinical Physiology, University Hospital, Getingevägen 4, SE-221 85 Lund, Sweden

## Abstract

**Introduction:**

Alveolar dead space reflects phenomena that render arterial partial pressure of carbon dioxide higher than that of mixed alveolar gas, disturbing carbon dioxide exchange. Right-to-left shunt fraction (Qs/Qt) leads to an alveolar dead space fraction (VdA_S_/VtA; where VtA is alveolar tidal volume). In acute respiratory distress syndrome, ancillary physiological disturbances may include low cardiac output, high metabolic rate, anaemia and acid-base instability. The purpose of the present study was to analyze the extent to which shunt contributes to alveolar dead space and perturbs carbon dioxide exchange in ancillary physiological disturbances.

**Methods:**

A comprehensive model of pulmonary gas exchange was based upon known equations and iterative mathematics.

**Results:**

The alveolar dead space fraction caused by shunt increased nonlinearly with Qs/Qt and, under 'basal conditions', reached 0.21 at a Qs/Qt of 0.6. At a Qs/Qt of 0.4, reduction in cardiac output from 5 l/minute to 3 l/minute increased VdA_S_/VtA from 0.11 to 0.16. Metabolic acidosis further augmented the effects of shunt on VdA_S_/VtA, particularly with hyperventilation. A Qs/Qt of 0.5 may increase arterial carbon dioxide tension by about 15% to 30% if ventilation is not increased.

**Conclusion:**

In acute respiratory distress syndrome, perturbation of carbon dioxide exchange caused by shunt is enhanced by ancillary disturbances such as low cardiac output, anaemia, metabolic acidosis and hyperventilation. Maintained homeostasis mitigates the effects of shunt.

## Introduction

In acute respiratory distress syndrome (ARDS), dead space is often high [1,2]. This impedes gas exchange and efforts to ventilate at low tidal volume in order to provide lung protective ventilation. Airway dead space is increased by connecting tubes, often including a humidifying filter, and by limiting time for equilibration between airway and alveolar space [3]. In a complex relationship, dead space at the alveolar level reflects uneven ventilation/perfusion among lung compartments. Ventilated compartments with nearly zero perfusion may result from microthrombosis. Other compartments may have a broad distribution of ventilation/perfusion relationships. In a ground breaking study, West [4] showed that this impedes gas exchange by increasing alveolar dead space.

In ARDS intrapulmonary shunt depends on collapsed lung units that are perfused but not ventilated. Part of venous blood thereby passes the lung without exchanging carbon dioxide and then mixes with arterial blood. Venous blood has a higher carbon dioxide content than does arterialized blood from ventilated and perfused lung units, and a shunt thereby leads to an increase in arterial carbon dioxide tension (PaCO_2_). Therefore, a right-to-left shunt widens the difference between alveolar carbon dioxide tension (PaCO_2_) and PaCO_2_, which defines the alveolar dead space (see Equation 1, below). Accordingly, it contributes to the classical concept physiological dead space [5,6]. Such a shunt may reach 50% of cardiac output or more and increases the need for alveolar ventilation (V'A) and total ventilation (V'tot) [7]. The effect of a shunt on arterial oxygenation is routinely considered in critical care and can easily be estimated by using the shunt equation [8]. The effect of shunt on dead space and carbon dioxide exchange reflects complex relationships between content and partial carbon dioxide tension and oxygen saturation in venous, arterial and pulmonary end-capillary blood. Applying a simplified lung model, Mecikalski and coworkers [9] calculated the extent to which shunt affects alveolar dead space under specific circumstances. Later, Giovannini and colleagues [10] developed a model that allows accurate calculations of difference in carbon dioxide concentration between venous and arterial blood. We amended this model to calculate effects of shunt on carbon dioxide exchange under different conditions.

The purpose of the present study was to analyze the extent to which intrapulmonary shunt contributes to alveolar dead space, thereby perturbing carbon dioxide exchange, at varying physiological conditions that are relevant in ARDS. The effects of varying cardiac output, metabolic rate, respiratory and metabolic acid-base status, haemoglobin concentration and haematocrit were analyzed, as were the effects of combinations of these factors.

## Materials and methods

**Figure 1:**
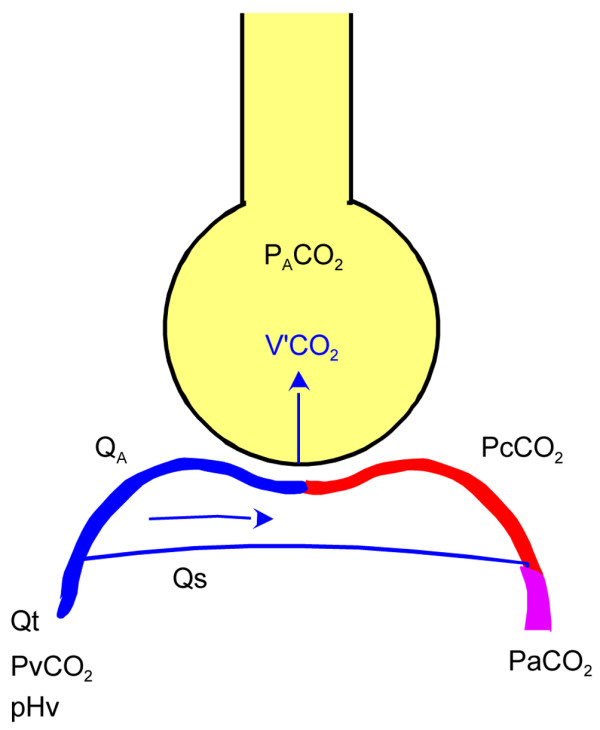
Simplified lung model. Total cardiac output (Qt) was distributed to ventilated capillaries (QA) and to a right-to-left shunt (Qs). At steady state the alveolar carbon dioxide tension (PaCO_2_) is the same as in the end-capillary blood (PcCO_2_). V'CO_2_, eliminated carbon dioxide (ml/minute); pHv, venous pH; PvCO_2_, venous carbon dioxide tension; PaCO_2_, arterial carbon dioxide tension.

Conventionally, the abbreviations used to denote partial pressure, saturation, content, arterial, venous, pulmonary end-capillary and alveolar include the letters P, S, C, a, v, c and A, respectively. Total cardiac output (Qt) is distributed to ventilated alveoli and shunt (blood flow to shunt [Qs]; Figure 1). Shunt fraction is denoted Qs/Qt.

At steady state, we assumed the following: equilibrium of diffusion between alveolar gas and pulmonary end-capillary blood and homogeneity of ventilation/perfusion among ventilated alveoli. Accordingly, PaCO_2 _was regarded to be equivalent to partial end-capillary carbon dioxide tension (PcCO_2_).

The part of alveolar dead space that is caused by shunt is denoted VdA_S_. The fraction of alveolar tidal volume (VtA) representing alveolar dead space caused by shunt (VdA_S_/VtA) was calculated using the following equation:

(1) VdA_S_/VtA = (PaCO_2 _- PaCO_2_)/PaCO_2 _= (PaCO_2 _- PcCO_2_)/PaCO_2_

PaCO_2 _and PcCO_2 _were determined for Qs/Qt from 0 to 0.6 by simulating various physiological conditions.

**Figure 2:**
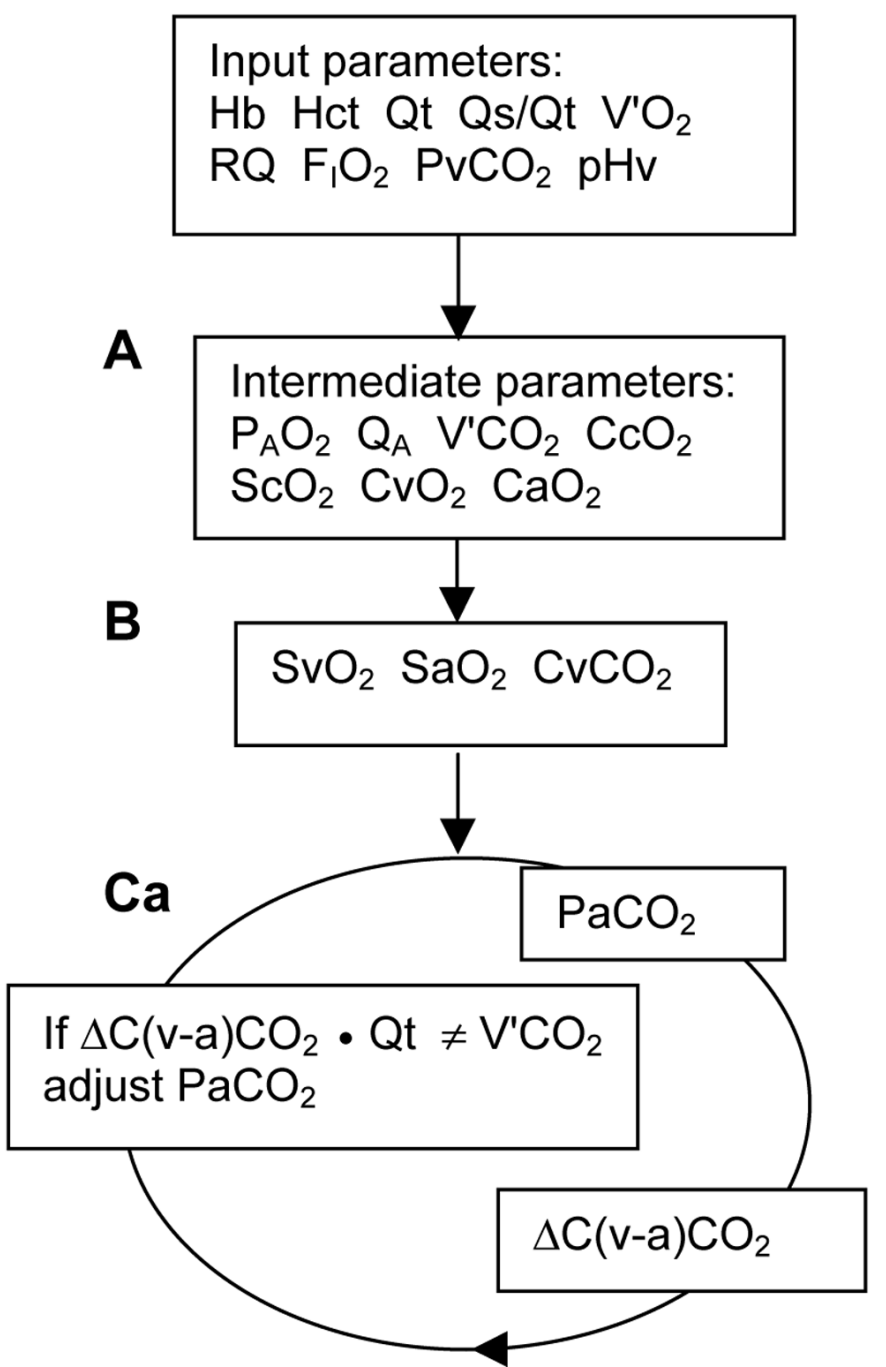
Outline of calculations further detailed in Additional file 1. **(a) **Starting out from input parameters, analytical calculations of intermediate parameters were performed using standard equations. **(b) **Input parameters, together with venous oxygen content (CvO_2_) and arterial oxygen content (CaO_2_), define unique values of venous oxygen saturation (SvO_2_) and arterial oxygen saturation (SaO_2_), which were iteratively determined. Venous carbon dioxide content (CvCO_2_) was calculated in accordance with the method reported by Giovannini and coworkers [10]. **(c) **In an extensive system of iterations, arterial carbon dioxide tension (PaCO_2_) was iteratively adjusted until veno-arterial difference in carbon dioxide content (ΔC [v-a]CO_2_) multiplied by total cardiac output (Qt) became equal to carbon dioxide elimination (V'CO_2_). In a step parallel to that shown in panel c, end-capillary carbon dioxide tension (PcCO_2_) was iteratively determined employing the value of QA (blood flow to ventilated alveoli) instead of Qt.

The simulation can in detail be followed in Additional file 1 and is outlined here with reference to Figure 2. Input parameters from which the simulation was initiated ('basal conditions') were as follows: haemoglobin 145 g/l, haematocrit 0.445, Qt 5 l/minute, oxygen consumption 250 ml/minute STPD (standard temperature and dry gas at standard barometric pressure), and respiratory quotient 0.8. Basal metabolic acid base balance was defined as venous pH 7.37, which according to Siggaard-Andersen [11] yields a base excess of zero. Venous carbon dioxide tension was for most simulations chosen so as to obtain a PaCO_2 _of 5.33 kPa. Fraction of inspired oxygen (FiO_2_) was increased from 0.4 to 0.7 or 1.0 to maintain an arterial oxygen saturation (SaO_2_) above 95%, if possible. Barometric pressure was 101.3 kPa, body temperature 37°C and the concentration of 2,3-diphosphoglycerate was 5 mmol/l in all simulations.

Intermediate parameters were calculated by adding 250 ml oxygen/minute to cardiac output and eliminating 200 ml carbon dioxide/minute from the same blood volume. For that we used the alveolar gas equation [12], equations describing the oxyhaemoglobin dissociation curve [13] and Fick's equation (Figure 2a).

Venous oxygen saturation was iteratively calculated from oxygen content in venous blood using the haemoglobin dissociation curve defined by input parameters. SaO_2 _was similarly derived. Venous carbon dioxide content (CvCO_2_) was calculated in accordance with the method reported by Giovannini and coworkers [10] (Figure 2b).

PaCO_2 _and PcCO_2 _were then obtained in order to calculate VdA_S_/VtA using Equation 1 (Figure 2c). This was done by simulating gas exchange in two iterated loops: one simulating the path from mixed venous blood to pulmonary end-capillary blood, and another simulating the path from mixed venous blood to arterial blood. The latter loop (Figure 2) began with an arbitrary, temporary PaCO_2_. The amount of carbon dioxide eliminated from cardiac output (Qt) while venous oxygen saturation changed to SaO_2 _and venous carbon dioxide tension changed to the temporary PaCO_2 _was calculated in accordance with Giovannini and coworkers. The temporary value of PaCO_2 _was iteratively adjusted until the calculated amount of carbon dioxide eliminated equalled 200 ml/minute (Solver in the Newton mode, Excel 2002; Microsoft Corp., Redmond, WA, USA). The other loop began with an arbitrary PcCO_2_; its value was iteratively adjusted until 200 ml carbon dioxide/minute was eliminated, but from the blood flowing through ventilated alveolar capillaries. VdA_S_/VtA was finally calculated using Equation 1. For each loop, iterations continued until difference from the desired carbon dioxide elimination was under 0.001 ml/minute. Carbon dioxide elimination that depended on increased oxygen saturation within the pulmonary capillaries (the Haldane effect) was separated from the carbon dioxide elimination that depended on reduction in carbon dioxide tension.

One purpose of ventilation is to effect carbon dioxide exchange and achieve and maintain the target PaCO_2_, whatever that may be. Accordingly, it may be necessary to increase V'A and V'tot in response to augmented VdA_S_/VtA. Alternatively, one may allow PaCO_2 _to increase. The VdA_S_/VtA values obtained from the simulations above with different Qs/Qt were used to calculate increases in V'A or PaCO_2 _(using Equations 2 to 4, below). Increases in V'tot were also calculated at constant PaCO_2_.

(2) PaCO_2 _= V'CO_2_/V'A × k = V'CO_2_/(RR × [Vt - Vd_phys_]) × k

(3) Vd_phys _= Vd_aw _+ VdA_VQ _+ VdA_S_

(4) VdA_S _= VdA_S_/VtA × (Vt - Vd_aw _- VdA_VQ_)

where V'CO_2 _is carbon dioxide elimination, k is barometric pressure, RR is the respiratory rate, Vt is the tidal volume, Vd_phys _is the physiological dead space, Vd_aw _is the airway dead space, and VdA_VQ _is the part of alveolar dead space that is caused by alterations in the ventilation/perfusion relationships other than shunt. The calculations were based upon the mean airway dead space of 0.2 l from the study conducted by Beydon and coworkers [1] and a deduced mean value for VdA_VQ _of 0.06 l from the same study. The calculations can be followed by reference to Additional file 1 (Figures 6 and 7 in Additional file 1).

## Results

**Figure 3:**
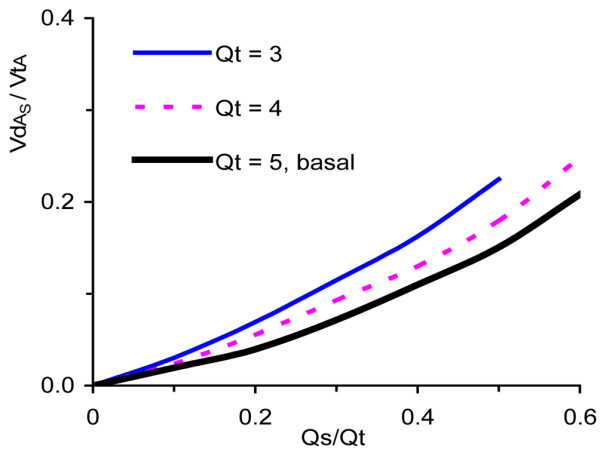
Alveolar dead space fraction versus shunt fraction at varying cardiac output. Shown is the alveolar dead space fraction (VdA_S_/VtA) versus shunt fraction (Qs/Qt) at varying cardiac output (Qt).

All iterative calculations efficiently met the convergence criterion. When, at basal conditions, Qs/Qt was increased to 0.5, VdA_S_/VtA reached 0.15 (Figure 3). At reduced Qt the VdA_S_/VtA paralleled the difference between CvCO_2 _and arterial carbon dioxide content (CaCO_2_); specifically, CvCO_2 _- CaCO_2 _increased from 40 ml/l at a Qt of 5 l/minute to 67 ml/l at a Qt of 3 l/minute. When CvCO_2 _- CaCO_2 _was increased to the same extent as when Qt was reduced, but via increased metabolic rate, the effects on VdA_S_/VtA were similar.

To maintain SaO_2 _at 95%, at basal conditions FiO_2 _was increased to 0.7 at a Qs/Qt of 0.3 and further to 1.0 at a Qs/Qt of 0.4. When FiO_2 _was increased to 1.0 at a Qs/Qt of 0.3, although SaO_2 _was above 95%, VdA_S_/VtA increased from 0.071 to 0.079. In contrast, if FiO_2 _was maintained at 0.4 while SaO_2 _fell to 91%, VdA_S_/VtA decreased to 0.063.

Normochromic anaemia (proportional decrease in haematocrit and haemoglobin) or hypochromic anaemia (constant haematocrit) was simulated by reducing haemoglobin from 145 to 97 and to 60 g/l. In both cases, at a Qs/Qt of 0.5 the VdA_S_/VtA increased from 0.15 to 0.17 and 0.19, respectively. Variation in haematocrit and respiratory quotient had only trivial effects on VdA_S_/VtA.

**Figure 4:**
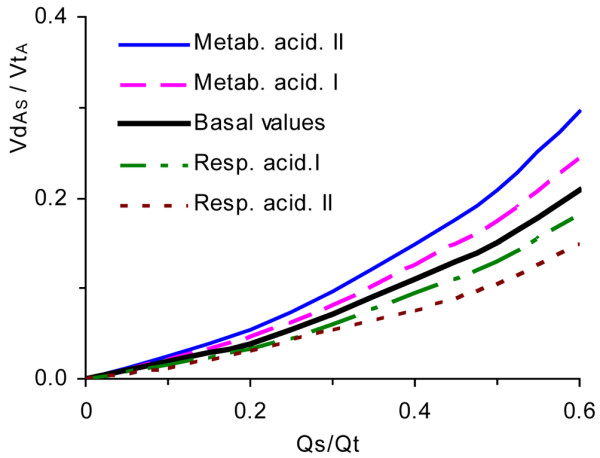
Alveolar dead space fraction versus shunt fraction at varying acid base status. Alveolar dead space fraction (VdA_S_/VtA) versus shunt fraction (Qs/Qt) at varying acid-base status. Respiratory acidosis I and II refer to arterial carbon dioxide tension (PaCO_2_) values of 9.1 kPa and 15.8 kPa, respectively, yielding arterial pH (pHa) values of 7.25 and 7.09, respectively. Metabolic acidosis I and II refer to base excess (BE) values of -9.0 mmol/l and -17 mmol/l, yielding pHa values of 7.25 and 7.10, respectively.

Respiratory acidosis, simulated by higher PaCO_2 _at zero base excess, led to lower VdA_S_/VtA (Figure 4). Metabolic acidosis had the opposite effect. For the conditions shown in Figure 4 and at a Qs/Qt of 0.4, the fraction of carbon dioxide exchange caused by the Haldane effect varied between 0.2 and 0.3. Low CvCO_2_, as occurs in metabolic acidosis or hyperventilation, was associated with low Haldane effect. At a Qs/Qt of 0.5, the VdA_S_/VtA correlated with the logarithm of CvCO_2 _(VdA_S_/VtA = -0.10 × ln [CvCO_2_] + 0.55; R = 0.997).

**Figure 5:**
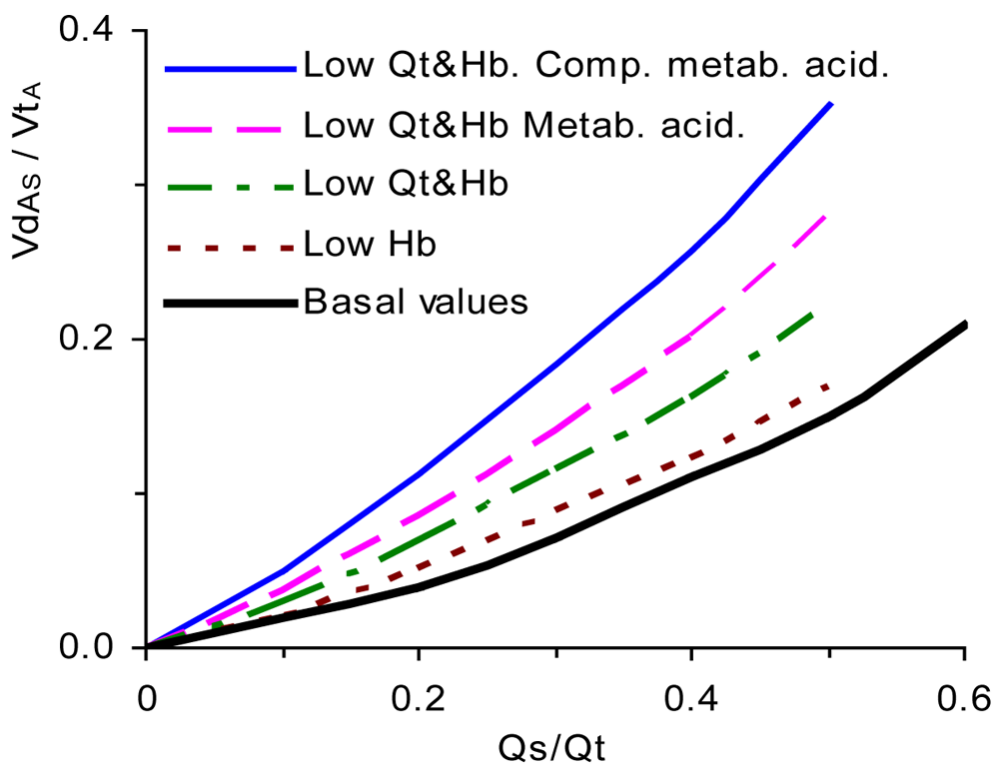
Alveolar dead space fraction versus shunt fraction at additive ancillary pathology. Alveolar dead space fraction (VdA_S_/VtA) versus shunt fraction (Qs/Qt) at additive ancillary pathology. Step-wise analyses of effects of a low haemoglobin (Hb; 97 g/l), low Hb and Qt (3.5 l/minute), low Hb and Qt and metabolic acidosis (base excess [BE] -13 mmol/l), and the latter case after respiratory compensation for acidosis by hyperventilation (arterial carbon dioxide tension [PaCO_2_] 2.1 kPa).

In critically sick patients, physiological aberrations are often combined. A patient in traumatic shock may have high Qs/Qt, and low haemoglobin and Qt. Tissue hypoxia may lead to metabolic acidosis. Figure 5 shows how VdA_S_/VtA would increase as a consequence of these successive or parallel phenomena. Particularly high values of VdA_S_/VtA were observed in compensated metabolic acidosis, characterized by hyperventilation to reduce PaCO_2 _so as to partially normalize pH.

More detailed data underlying Figures 3 to 5 are presented in Additional file 1.

**Figure 6:**
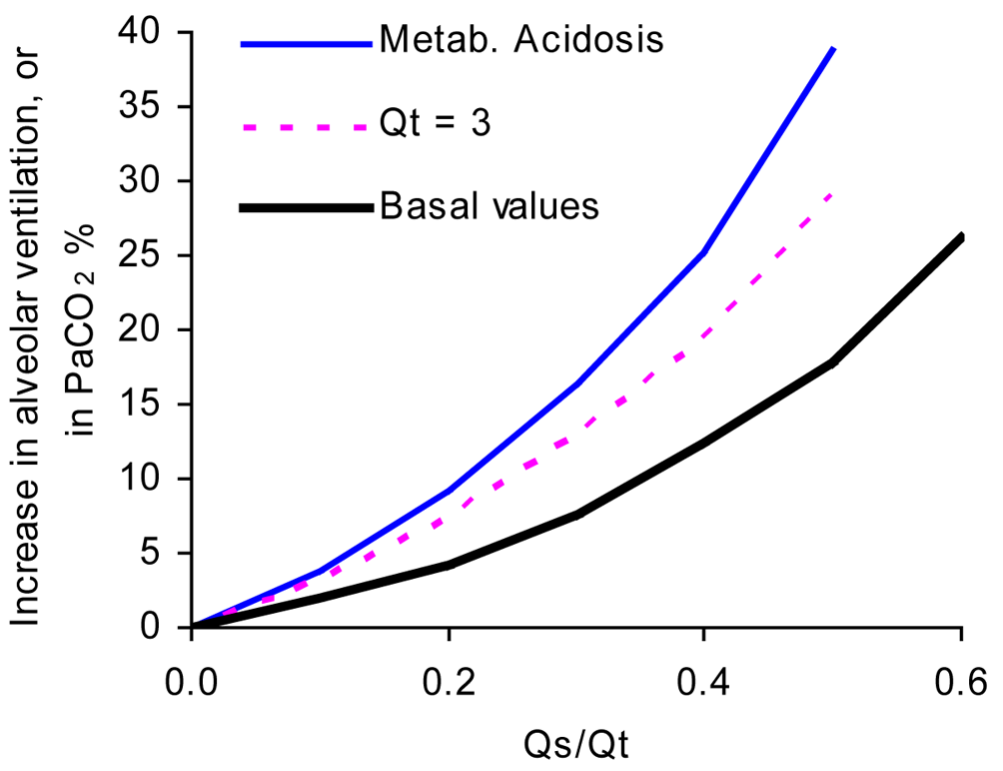
Increase in PaCO_2 _or alveolar ventilation versus shunt fraction. Increase in arterial carbon dioxide tension (PaCO_2_; %) at constant alveolar ventilation versus shunt fraction. This is equivalent to required increase in alveolar ventilation to maintain PaCO_2_. Examples are as follows: 'Basal': Qt = 5 l/minute, haemoglobin (Hb) = 145 g/l and base excess (BE) = 0; 'Qt = 3': Qt = 3 l/minute, Hb = 145 g/l and BE = 0; and 'Metab. acidosis': Qt = 3.5 l/minute, Hb = 97 g/l and BE = -13.

**Figure 7:**
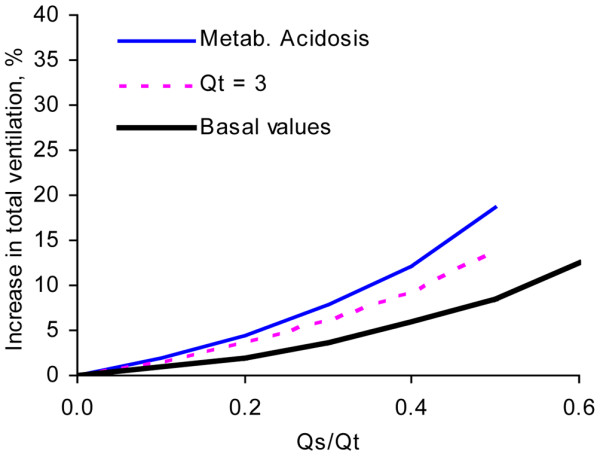
Increase in total ventilation versus shunt fraction at constant PaCO_2_. Required increase in total ventilation (%) at different shunt fractions (Qs/Qt) to maintain arterial carbon dioxide tension (PaCO_2_) constant. Examples are as follows: 'Basal': Qt = 5 l/minute, haemoglobin (Hb) = 145 g/l and base excess (BE) = 0; 'Qt = 3': Qt = 3 l/minute, Hb = 145 g/l and BE = 0; and 'Metab. acidosis': Qt = 3.5 l/minute, Hb = 97 g/l and BE = -13. Airway dead space (Vd_aw_) and the alveolar dead space caused by uneven ventilation/perfusion (VdA_VQ_) were assumed to be 0.2 l and 0.06 l, respectively.

Depending upon which strategy is chosen to balance gas exchange with lung protection, one can increase ventilation or allow PaCO_2 _to increase in response to the effect of shunt. At basal conditions, at reduced Qt (3 l/minute) and at reduced Qt combined with low haemoglobin and metabolic acidosis, a Qs/Qt of 0.5 would result in increases in V'A (or in PaCO_2_) of 18%, 29% and 39%, respectively (Figure 6). Regarding dead space of a non-VdA_S _origin, the increase in total ventilation needed to maintain PaCO_2 _would be 8.5%, 14% and 19% at the same conditions as above (Figure 7). In the setting of a tidal volume of 450 ml and a respiratory rate of 20 breaths/minute, a Qs/Qt of 0.5 would be accompanied by increases in tidal volume by 38 ml, 62 ml and 84 ml for the three conditions.

## Discussion

The present study focuses on one of many factors to consider when balancing adequate gas exchange with minimal ventilator-induced lung injury during mechanical ventilation in ARDS patients (alveolar dead space related to intrapulmonary shunt). The rationale underpinning this approach is that increased understanding of all such factors may form the basis for improved treatment, and not only with respect to how the patient should be ventilated. The effect on dead space of shunt was studied under varied physiological circumstances, such that may occur in ARDS; notable among these are variation in metabolic rate, respiratory quotient, cardiac output, haemoglobin and acid base status. By incorporating into our model the parameters reported by Mecikalski and coworkers [9], we were largely able to corroborate their findings, although our values for VdA_S_/VtA are slightly higher. However, in relation to the work conducted by Mecikalski and coworkers [9], our findings regarding the effects of variation in haemoglobin, acid base status and combinations of physiological aberrations are novel. Additional file 1 can be used to verify and expand upon the results by entering alternative input parameters. The study did not incorporate diffusion limitation or uneven ventilation/perfusion – factors that are more important than shunt in other groups of critically ill patients.

Our analysis of VdA_S_/VtA was based upon well validated equations, which together describe the highly complex process of gas exchange. We employed iterative mathematics, as first applied by West [4], and the algorithms developed by Giovannini and coworkers [10] allow modelling of carbon dioxide exchange with particular precision.

Transport of carbon dioxide and the mechanisms underlying its turnover depend on several factors, each of which are mediated by nonlinear relationships between two or more factors. The physiological background to the effects of shunt on dead space and on carbon dioxide exchange at differing physiological conditions is therefore complex. In each situation it is nevertheless possible to recognize the primary mechanism underlying the effects of shunt. A low cardiac output or a high metabolic rate augments the venous content of carbon dioxide, and thereby the effect on shunt on PaCO_2_. The effect of anaemia can be attributed to the fact that fewer haemoglobin molecules are available to absorb the excess carbon dioxide transferred to arterialized blood via shunted blood. Therefore, in a state of anaemia, this excess will to an increased extent appear as dissolved carbon dioxide. This leads to an enhanced increase in PaCO_2_. A high FiO_2 _increases oxygen content and saturation in blood from ventilated lung compartments and in arterial blood. Through the Haldane effect, haemoglobin will then carry less carbon dioxide, leading to a surplus that will be carried as dissolved carbon dioxide, thus increasing PaCO_2_. In acid-base perturbations, the effects of shunt on VdA_S_/VtA were tightly and negatively related to ln(CvCO_2_). At low CvCO_2_, such as occurs in respiratory alcalosis, the Haldane effect is less efficient. This hampers alveolar carbon dioxide exchange and contributes to alveolar dead space.

Effects on VdA_S_/VtA of shunt fractions up to about 0.2 to 0.3 are small and are of minimal clinical significance. Higher degrees of shunt, particularly when combined with complicating physiological aberrations, the effect of shunt on carbon dioxide exchange merits attention. An example is metabolic acidosis combined with hyperventilation. Increased VdA_S_/VtA should be added to known harmful effects of hyperventilation.

Clinically relevant effects of increasing VdA_S_/VtA are permissively increased PaCO_2 _or, equivalently, increased alveolar ventilation (Figure 6). Obviously, one may choose a compromise between these two alternatives. Figure 7 shows that total ventilation at high shunt fraction may need to be increased by 10% to 20%, depending upon concurrent pathophysiology. This estimate was based upon values for other dead space compartments regarded as typical for ARDS. One may reason that this is an effect of limited clinical importance. On the other hand, an awareness of all of factors that are of importance to the magnitude of dead space fractions may allow us to develop less traumatic ventilation strategies. Clearly, airway dead space caused by connecting tubes and humidifiers is one such factor. Mode of inspiration is another; dead space can also be reduced by selecting a mode of inspiration that lengthens the mean distribution time during which the alveolar tidal volume is present in the respiratory zone [3,14].

The essence of intensive care is to support the patient by maintaining homeostasis. In ARDS, adequate oxygenation may be achieved by reducing intrapulmonary shunt using ventilation patterns that favour lung recruitment [15,16]. Such strategies have the additional benefits of reducing VdA_S_/VtA and the associated perturbation in carbon dioxide exchange. Other routines in intensive care serve to maintain adequate cardiac output, to control metabolic rate, and to avoid anaemia and to maintain a proper acid base balance. All of them lead to lower VdA_S_/VtA and reduced requirements for ventilation. A high FiO_2 _may lead to toxicity and enhances alveolar derecruitment in ARDS [17]. As shown, an unduly high FiO_2 _also augments alveolar dead space.

This study provides additional motivation to maintain homeostasis in ARDS. It underscores how combinations of physiological aberrations may lead to inefficient carbon dioxide exchange related to intrapulmonary shunting of blood.

## Conclusion

In ARDS, perturbation of carbon dioxide exchange caused by high shunt fraction is enhanced by ancillary disturbances such as low cardiac output, anaemia, metabolic acidosis and hyperventilation. Maintained homeostasis mitigates the effects of shunt.

## Key messages

• In ARDS intrapulmonary shunt perturbs carbon dioxide exchange by increasing alveolar dead space, particularly in the presence of low cardiac output, reduced haemoglobin levels and metabolic acidosis.

• Maintained homeostasis mitigates these effects of shunt.

## Abbreviations

ARDS = acute respiratory distress syndrome; CvCO_2 _= venous carbon dioxide content; FiO_2 _= fraction of inspired oxygen; PaCO_2 _= arterial carbon dioxide tension; PaCO_2 _= alveolar carbon dioxide tension; PcCO_2 _= partial end-capillary carbon dioxide tension; Qs = blood flow to shunt; Qs/Qt = shunt fraction; Qt = total cardiac output; SaO_2 _= arterial oxygen saturation; VdA_S _= alveolar dead space caused by shunt; VdA_VQ _= alveolar dead space caused by uneven ventilation/perfusion; V'A = alveolar ventilation; VtA = alveolar tidal volume; V'tot = total ventilation.

## Competing interests

The authors declare that they have no competing interests.

## Authors' contributions

JE conducted preliminary analyses. LN and BJ together developed the calculation program, performed the analyses and wrote the manuscript. All authors read and approved the final manuscript.

## Supplementary Material

Additional file 1 Dead space caused by shunt. This executable Excel file allows calculation of alveolar dead space fraction caused by shunt under different physiological conditions. Click here for file

## References

[B1] Beydon L, Uttman L, Rawal R, Jonson B (2002). Effects of positive end-expiratory pressure on dead space and its partitions in acute lung injury. Intensive Care Med.

[B2] Nuckton TJ, Alonso JA, Kallet RH, Daniel BM, Pittet JF, Eisner MD, Matthay MA (2002). Pulmonary dead-space fraction as a risk factor for death in the acute respiratory distress syndrome. N Engl J Med.

[B3] Åström E, Uttman L, Niklason L, Aboab J, Brochard L, Jonson B (2008). Pattern of inspiratory gas delivery affects CO_2 _elimination in health and after acute lung injury. Intensive Care Med.

[B4] West JB (1969). Ventilation-perfusion inequality and overall gas exchange in computer models of the lung. Respir Physiol.

[B5] Fletcher R, Jonson B, Cumming G, Brew J (1981). The concept of deadspace with special reference to the single breath test for carbon dioxide. Br J Anaesth.

[B6] West JB, Jones NL (1965). Effects of changes in topographical distribution of lung blood flow on gas exchange. J Appl Physiol.

[B7] Eriksson L, Wollmer P, Olsson CG, Albrechtsson U, Larusdottir H, Nilsson R, Sjögren A, Jonson B (1989). Diagnosis of pulmonary embolism based upon alveolar dead space analysis. Chest.

[B8] Riley RL, Permutt S (1973). Venous admixture component of the AaPO_2 _gradient. J Appl Physiol.

[B9] Mecikalski MB, Cutillo AG, Renzetti AD (1984). Effect of right-to-left shunting on alveolar dead space. Bull Eur Physiopathol Respir.

[B10] Giovannini I, Chiarla C, Boldrini G, Castagneto M (1993). Calculation of venoarterial CO_2 _concentration difference. J Appl Physiol.

[B11] Siggaard-Andersen O (1963). Blood acid-base alignment nomogram. Scand J Clin Lab Invest.

[B12] Riley RL, Lilienthal JLJ, Proemmel D, Franke RE (1948). On the determination of the physiologically effective pressures of oxygen and carbon dioxide in alveolar air. Am J Physiol.

[B13] Siggaard-Andersen O, Wimberley PD, Fogh-Andersen N, Giovannini I, Gøthgen IH (1988). Measured and derived quantities with modern pH and blood gas equipment: calculation algorithms with 54 equations. Scand J Clin Lab Invest.

[B14] Aboab J, Niklason L, Uttman L, Kouatchet A, Brochard L, Jonson B (2007). CO_2 _elimination at varying inspiratory pause in acute lung injury. Clin Physiol Funct Imaging.

[B15] Richard JC, Brochard L, Vandelet P, Breton L, Maggiore SM, Jonson B, Clabault K, Leroy J, Bonmarchand G (2003). Respective effects of end-expiratory and end-inspiratory pressures on alveolar recruitment in acute lung injury. Crit Care Med.

[B16] Richard JC, Maggiore SM, Jonson B, Mancebo J, Lemaire F, Brochard L (2001). Influence of tidal volume on alveolar recruitment. Respective role of PEEP and a recruitment maneuver. Am J Respir Crit Care Med.

[B17] Aboab J, Jonson B, Kouatchet A, Taille S, Niklason L, Brochard L (2006). Effect of inspired oxygen fraction on alveolar derecruitment in acute respiratory distress syndrome. Intensive Care Med.

